# Evaluation of tumour hypoxia during radiotherapy using [^18^F]HX4 PET imaging and blood biomarkers in patients with head and neck cancer

**DOI:** 10.1007/s00259-016-3429-y

**Published:** 2016-06-01

**Authors:** Catharina M. L. Zegers, Frank J. P. Hoebers, Wouter van Elmpt, Judith A. Bons, Michel C. Öllers, Esther G. C. Troost, Daniëlle Eekers, Leo Balmaekers, Marlies Arts-Pechtold, Felix M. Mottaghy, Philippe Lambin

**Affiliations:** 1 Department of Radiation Oncology (MAASTRO), GROW – School for Oncology and Developmental Biology, Maastricht University Medical Centre, Maastro Clinic, Dr. Tanslaan 12, 6229ET Maastricht, The Netherlands; 2 Central Diagnostic Laboratory, Maastricht University Medical Centre, Maastricht, The Netherlands; 3 Helmholtz Zentrum Dresden-Rossendorf, Dresden, Germany; 4 OncoRay, Department of Radiation Oncology, Medical Faculty and University Hospital Carl Gustav Carus, Technische Universität Dresden, Dresden, Germany; 5 Department of Nuclear Medicine, Maastricht University Medical Centre, Maastricht, The Netherlands; 6 Department of Nuclear Medicine, RWTH Aachen University, University Hospital, Aachen, Germany

**Keywords:** Hypoxia, PET, CAIX, Osteopontin, VEGF

## Abstract

**Background and purpose:**

Increased tumour hypoxia is associated with a worse overall survival in patients with head and neck squamous cell carcinoma (HNSCC). The aims of this study were to evaluate treatment-associated changes in [^18^F]HX4-PET, hypoxia-related blood biomarkers, and their interdependence.

**Material and methods:**

[^18^F]HX4-PET/CT scans of 20 patients with HNSCC were acquired at baseline and after ±20Gy of radiotherapy. Within the gross-tumour-volumes (GTV; primary and lymph nodes), mean and maximum standardized uptake values, the hypoxic fraction (HF) and volume (HV) were calculated. Also, the changes in spatial uptake pattern were evaluated using [^18^F]HX4-PET/CT imaging. For all patients, the plasma concentration of CAIX, osteopontin and VEGF was assessed.

**Results:**

At baseline, tumour hypoxia was detected in 69 % (22/32) of the GTVs. During therapy, we observed a significant decrease in all image parameters. The HF decreased from 21.7 ± 19.8 % (baseline) to 3.6 ± 10.0 % (during treatment; *P* < 0.001). Only two patients had a HV > 1 cm^3^ during treatment, which was located for >98 % within the baseline HV. During treatment, no significant changes in plasma CAIX or VEGF were observed, while osteopontin was increased.

**Conclusions:**

[^18^F]HX4-PET/CT imaging allows monitoring changes in hypoxia during (chemo)radiotherapy whereas the blood biomarkers were not able to detect a treatment-associated decrease in hypoxia.

**Electronic supplementary material:**

The online version of this article (doi:10.1007/s00259-016-3429-y) contains supplementary material, which is available to authorized users.

## Introduction

Tumour cell hypoxia is known to promote resistance to cancer treatment, to increase tumour aggressiveness, and to be a prognostic factor for survival [1]. Non-invasive imaging of tumour hypoxia by means of positron emission tomography (PET) has been shown to predict loco-regional control and survival, and may be used to select patients for additional anti-hypoxia therapy [2–4]. In addition, PET imaging can be used to monitor the response to treatment. Previous studies using the hypoxia PET tracer [^18^F]FMISO and the metabolic PET tracer [^18^F]FDG observed that the uptake changes (early) during (chemo)radiotherapy had a higher predictive value than pre-treatment measurements [3, 5]. Previously, Overgaard [6] showed that the modification of hypoxia during radiotherapy results in better loco-regional control and survival in patients with a squamous cell carcinoma of the head and neck (HNSCC). However, stratifying patients undergoing ARCON (accelerated radiotherapy with carbogen and nicotinamide) based on their pre-therapeutic hypoxic status (pimonidazole staining) demonstrated that the benefit in loco-regional control was specifically observed for patients with initial tumour hypoxia before the start of treatment [7, 8]. The pimonidazole staining of a biopsy, however, can only provide information about local hypoxia before the start of treatment. Non-invasive hypoxia PET imaging, on the other hand, provides an opportunity to perform repeated tumour hypoxia measurements in 3-dimensions. Therefore hypoxia PET measurements may be used to select patients likely to have a benefit from additional anti-hypoxia therapy.

One of the hypoxia PET tracers to visualize and quantify tumour hypoxia is the 2-nitroimidazole3-[^18^F]fluoro-2-(4-((2-nitro-1H-imidazol-1-yl)methyl)-1H-1,2,3-triazol-1-yl)propan-1-ol, [^18^F]HX4. In previous pre-clinical studies [^18^F]HX4 was validated as a hypoxia tracer, and the repeatability of the tracer uptake was assessed [9, 10]. In addition, in patients with non-small cell lung cancer (NSCLC), [^18^F]HX4 showed promising results and was shown to provide additional value to the metabolic PET tracer [^18^F]FDG [11, 12]. In this paper, we will investigate the potential of this tracer to detect treatment-associated changes in hypoxic tumour status in patients with HNSCC.

Another method to obtain information on tumour hypoxia may be the measurement of hypoxia-related proteins or enzymes in plasma. Potential relevant hypoxia markers are plasma osteopontin, carbonic anhydrase IX (CAIX), and vascular endothelial growth factor (VEGF). Osteopontin is activated under hypoxic conditions and is inversely correlated with the PO_2_ value of the tumour [13]. In addition, plasma osteopontin is a significant predictor for the response to radiotherapy in patients with head and neck cancer [14]. CAIX expression is upregulated under the influence of tumour hypoxia. Also, in patients with NSCLC, a high level of plasma CAIX was associated with a shorter overall survival [15]. Last, hypoxia activates hypoxia inducible factor (HIF-1), which induces a high expression of VEGF, the primary cytokine related to angiogenesis. VEGF may, therefore, serve as an indirect marker of tumour hypoxia. These three markers might have the potential to stratify patients based on their hypoxic tumour status.

The aims of this study were to evaluate the changes in hypoxia during treatment in patients with HNSCC, the spatial stability of the uptake pattern, and the presence of plasma osteopontin, CAIX, and VEGF in relationship to hypoxia imaging.

## Materials and methods

### Patients

Between January 2012 and October 2014, we included 20 patients in this imaging study. Patient, tumour, and treatment characteristics are shown in Table 1. The study was approved by the Ethical Review Committee of Maastricht University Medical Centre and registered on clinicaltrial.gov (NCT01347281). All patients provided written informed consent before study entry.

**Table 1:** Patient, tumour, and treatment characteristics

	N	%
Gender		
Male	17	85
Female	3	15
Pathology		
Squamous cell carcinoma	20	100
Tumour site		
Oropharynx	7	35
Larynx	8	40
Hypopharynx	5	25
HPV status (Oropharynx)		
Positive	3	43
Negative	3	43
Unknown	1	14
cT-Stage		
T1	1	5
T2	6	30
T3	11	55
T4	2	10
cN-Stage		
N0	9	45
N1	3	15
N2a	1	5
N2b	7	35
Stage grouping		
Stage II	2	10
Stage III	8	40
Stage IV	10	50
Treatment		
Radiotherapy	6	30
Cisplatin chemo-radiotherapy	10	50
Cetuximab-radiotherapy	4	20
[^18^F]HX4 PET imaging		
Baseline	20	100
During RT	17	85
Radiotherapy dose between [^18^F]HX4 scans [Gy]		
18	3	18
20	5	29
22	6	35
24	2	12
26	1	6

### PET/CT imaging

[^18^F]HX4 was produced as described in previous publications [9, 16–18]. After intravenous administration of an average (±SD) dose of 378 ± 84 MBq [^18^F]HX4, PET/CT imaging was performed at 1.5, 3, and 4 h post-injection (p.i.) for 15, 15, and 20 min, respectively, for a single bed position centred around the primary tumour. After ten patients, an interim analysis showed highest contrast at the imaging time-point at 4 h p.i., and this was used from then onwards.

[^18^F]HX4 PET/CT scans were acquired before the start of external beam radiotherapy and during the radiation treatment; after an average (±SD) dose of 21 ± 2 Gy using a Biograph 40 PET/CT scanner (Siemens Healthcare, Erlangen, Germany). Scatter and attenuation corrections were applied, PET images were reconstructed using OSEM 2D (Ordered Subset Expectation Maximization, four iterations, eight subsets) and a Gaussian filter of 5 mm. Imaging was performed in radiotherapy position, with the patient positioned on a flat table top using an immobilization mask and a movable laser alignment system.

### Image analysis

The gross tumour volume of the primary lesion (GTV_prim_) and involved lymph nodes (GTV_ln_), were delineated on the [^18^F]FDG PET/CT, used for radiotherapy planning purposes, by two experienced radiation oncologists in consensus. These contours were copied to the [^18^F]HX4 PET scan at baseline and during treatment by rigid registration. Maximum and mean standardized uptake values (SUV_max_ and SUV_mean_), were determined within the GTVs. In addition, the maximum tumour-to-muscle ratio (TMR_max_), was calculated, defined as the SUV_max_ in the tumour divided by the SUV_mean_ in the trapezius muscles (Supplementary Fig. 1). The hypoxic fraction (HF) and hypoxic volume (HV) were defined as the fraction or volume of the GTV with a TMR larger than 1.4.

To evaluate the spatial location of the [^18^F]HX4 PET uptake at baseline and during treatment, the [^18^F]HX4 PET/CT acquired during radiotherapy was rigidly registered to the baseline [^18^F]HX4 PET/CT. The rigid transformation was determined by the registration of the CT scans; subsequently, the same transformation was applied to the PET scans and GTV to co-register the images. A visual and voxel-wise comparison of the [^18^F]HX4 uptake before and during radiotherapy was performed to compare spatial uptake patterns for both GTV_prim_ and GTV_ln_.

### Blood biomarker analysis

For all patients, blood samples were collected before and during (chemo)radiotherapy on the day of the [^18^F]HX4 PET/CT scan. All blood biomarkers were measured in EDTA plasma. Samples were analysed simultaneously in a certified laboratory, using commercially available kits. CAIX was measured by a sandwich-type immunoassay that uses a mouse monocolonal capture antibody (V10) and a biotinylated mouse monocolonal antibody (M75) as detector (Nuclea Diagnostic Laboratories LLC, Cambridge, MA). Osteopontin and VEGF were measured by an ELISA method. A monoclonal antibody specific for osteopontin/VEGF was pre-coated onto the microplate and an enzyme-lined polyclonal antibody specific for osteopontin /VEGF was used as detector (R&D Systems (Minneapolis, MN).

To compare the plasma hypoxia markers with the [^18^F]HX4 uptake in the GTV_prim_ and GTV_ln_, we hypothesize that these markers reflect the uptake in all lesions within one patient. Therefore, the image parameters of multiple GTVs were combined, providing one SUV_max_ and TMR_max_ (the maximum of the present lesions). The hypoxic volumes were summed and for the SUV_mean_ a weighted average was calculated using:

$$ weighted\ SU{V}_{mean} = \frac{{\mathrm{SUV}}_{\mathrm{mean}\ \mathrm{GTVprim}}\ *\ {\mathrm{GTV}}_{\mathrm{prim}} + {\mathrm{SUV}}_{\mathrm{mean}\ \mathrm{GTVln}}\ *\ {\mathrm{GTV}}_{\ln }}{{\mathrm{GTV}}_{\mathrm{prim}} + {\mathrm{GTV}}_{\ln }} $$

### Statistical analysis

For all parameters, mean ± 1 standard deviation (SD) are reported. Non-parametric tests were used to determine significant differences in image and blood plasma parameters (Wilcoxon signed rank test) and to evaluate correlations between imaging parameters and blood parameters (Spearman’s correlation coefficient; R_s_). Linear regressions were performed to quantify the voxel-wise comparison, and a Pearson correlation coefficient (R_p_) was calculated. A *p*-value < 0.05 was assumed to be statistically significant.

## Results

In this study we analysed the [^18^F]HX4 uptake of 20 patients with HNSCC before the start of radiotherapy. For 3/20 patients the [^18^F]HX4 PET scan during radiotherapy was not performed due to the patient’s preference or health status. Eleven patients had involvement of the lymph nodes (GTV_ln_), which were separately analysed from the primary lesion (GTV_prim_). The average lesion sizes for GTV_prim_ was 17.6 ± 12.3 cm^3^ (range: 2.4–46.6 cm^3^) and for GTV_ln_ 22.6 ± 30.5 (range: 1.3–105.2 cm^3^).

### [^18^F]HX4 PET uptake at baseline

**Fig. 1:**
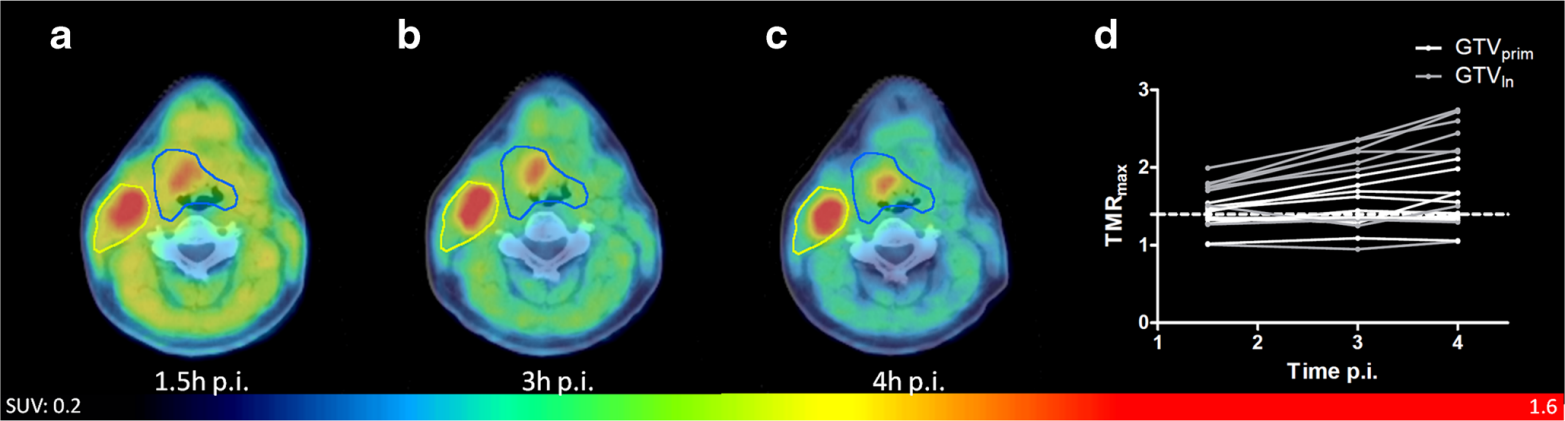
[^18^F]HX4 PET/CT scans of a patient with a T2N2bMx squamous cell carcinoma of the oropharynx, scanned at 1.5 h (*a*), 3 h (**b**), and 4 h p.i. (**c**). **d**: The tumour to muscle ratio (TMR_max_) for all patients. Shown are the gross tumour volumes of the primary lesions (GTV_prim_) and the metastatic lymph nodes (GTV_ln_), which increased significantly (1.5 h vs 3 h: *p* < 0.01, 3 h vs 4 h: *p* = 0.02)

In the first ten patients, [^18^F]HX4 PET/CT imaging was acquired at 1.5, 3, and 4 h p.i. In their lesions (10 GTV_prim_ and 9 GTV_ln_) we observed a significant increase in the TMR_max_ from 1.5 h (1.5 ± 0.3) to 3 h p.i. (1.7 ± 0.4; *P* < 0.01), and from 3 to 4 h p.i. (1.8 ± 0.6; *P* = 0.02) (Fig. 1). Therefore, [^18^F]HX4 PET/CT imaging at 4 h p.i. was selected as the standard and applied as the single imaging timepoint for the remaining patients.

At baseline we observed tumour hypoxia (TMR > 1.4 at 4 h p.i.) in 69 % (22/32) of the GTV_prim_ and GTV_ln_. For all lesions, we observed an average SUV_mean_, SUV_max_ and TMR_max_ of 0.8 ± 0.2, 1.3 ± 0.5 and 1.7 ± 0.5, respectively. The average HF and HV were 16 ± 20 % and 4.9 ± 9.6 cm^3^. All these image parameters were significantly correlated to the volume of the lesion, SUV_mean_ (R_s_ = 0.38, *p* = 0.03), SUV_max_ (R_s_ = 0.57, *p* < 0.001), TMR_max_ (R_s_ = 0.75, *p* < 0.001), HF (R_s_ = 0.63, *p* < 0.001) and HV (R_s_ = 0.74, *p* < 0.001).

### [^18^F]HX4 PET uptake during treatment

**Fig. 2:**
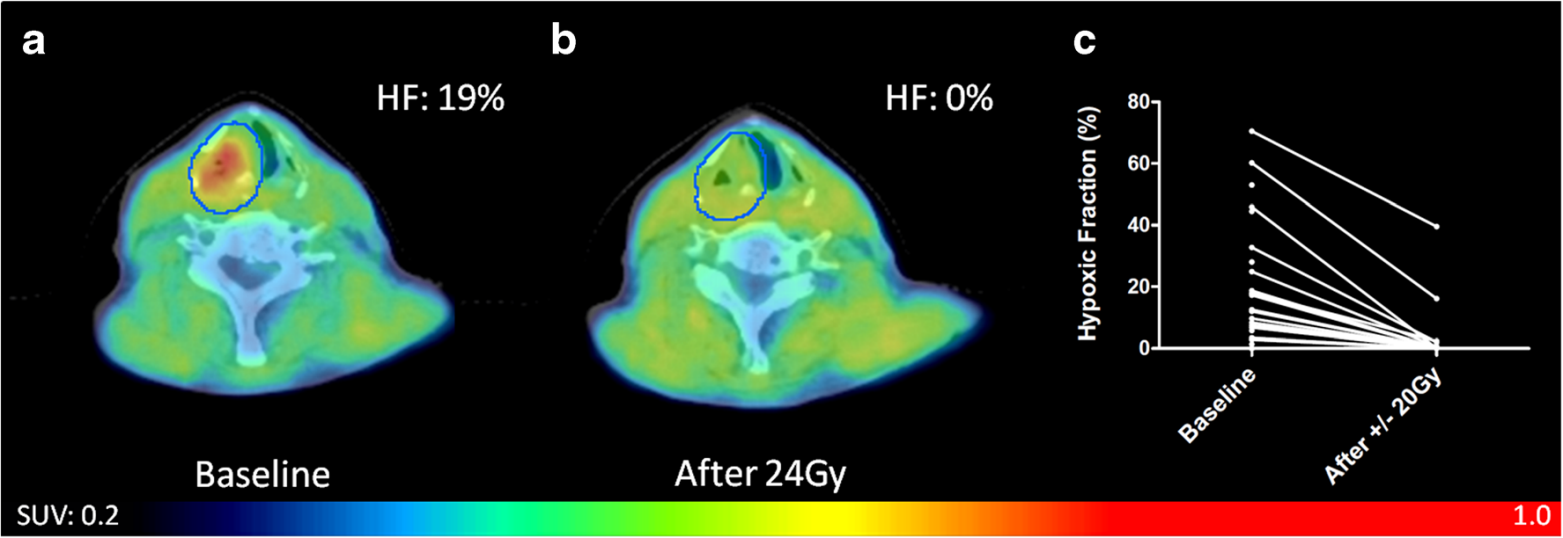
[^18^F]HX4 PET/CT scan of a patient with a T3N2bMx squamous cell carcinoma of the hypopharynx treated with cisplatin chemo-radiation. **a**: Scan with hypoxic primary tumour at baseline, **b**: decreased level of hypoxia during treatment and **c**: Calculated hypoxic fraction (HF) of all primary tumours and lymph nodes before and during treatment, significant decrease (*p* < 0.001)

We observed a significant correlation between the image parameters measured at baseline and during treatment: SUV_mean_ (R_s_ = 0.66, *P* < 0.001), SUV_max_ (R_s_ = 0.63, *P* < 0.001), TBR_max_ (R_s_ = 0.57, *P* < 0.01), HF (R_s_ = 0.56, *P* < 0.01), HV(*R* = 0.52, *P* < 0.01). Taking into account the hypoxic lesions (GTV_prim_ and GTV_ln_), with a [^18^F]HX4 PET/CT scan at baseline and during treatment (*N* = 17), we observed a significant decrease in all image-derived parameters during therapy (Table 2; Fig. 2). This decrease was independent of the given treatment (Supplementary Table 1). Of the 17 hypoxic lesions at baseline, only seven had a HF > 0 during treatment. In the other ten lesions hypoxia as measured by [^18^F]HX4 PET imaging had disappeared.

**Table 2:** [^18^F]HX4 PET/CT parameters at baseline and during therapy. Shown are the mean, standard deviation, range, and the percentage difference

	Baseline	During treatment	Difference [%]	Significance (*P*-value)
SUV_mean_	0.9 ± 0.2 (0.7–1.3)	0.8 ± 0.2 (0.5–1.2)	−13 ± 19	0.02
SUV_max_	1.5 ± 0.4 (0.9–2.1)	1.1 ± 0.3 (0.7–1.9)	−25 ± 18	0.001
TMR_max_	1.9 ± 0.4 (1.4–2.8)	1.4 ± 0.2 (1.0–2.1)	−27 ± 11	<0.001
Hypoxic fraction [%]	22 ± 20 (3–71)	4 ± 10 (0–40)	−93 ± 15	<0.001
Hypoxic volume [cm^3^]	4.6 ± 5.2 (0.1–18.0)	0.8 ± 2.5 (0.0–10.1)	−93 ± 15	<0.001

Shown are the baseline hypoxic lesions (GTV_prim_ and GTV_ln_), with an [^18^F]HX4 PET/CT scan at baseline and during treatment (total lesions *N* = 17). The provided significance is based on the Wilcoxon signed rank test

Only one non-hypoxic lesion (TBR = 1.37, HF = 0) at baseline, changed its hypoxic status during treatment. However, The TBR_max_ increased only from 1.37 at baseline to 1.43 during treatment resulting in a small HF and HV during treatment of 0.4 % and 0.2 cm^3^, respectively.

### Spatial stability of [^18^F]HX4 PET uptake

**Fig. 3:**
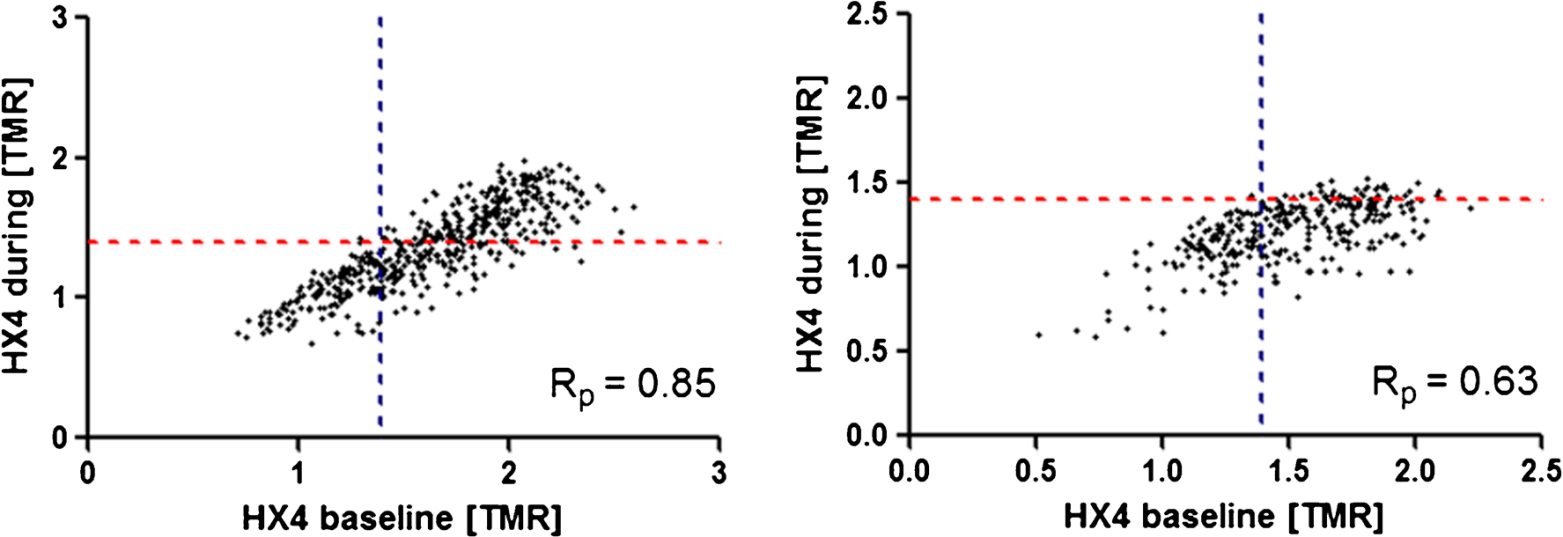
Spatial reproducibility of the [^18^F]HX4 PET uptake in two patients with persistent hypoxia during treatment (*left*: patient with cT2N2aM0 hypopharynx cancer, *right*: patient with cT2N2bM0 oropharynx cancer. The PET-CT scans during treatment were in both patients performed after 18 Gy. Visualised is the voxel-wise correlation of the [^18^F]HX4-uptake within the GTV

To perform an analysis of the spatial [^18^F]HX4 uptake, a significant tracer uptake in both [^18^F]HX4 PET/CT scans was necessary. Only two lesions (both GTV_ln_) had a HV larger than 1 cm^3^ during treatment (2.9 and 10.1 cm^3^). These GTV_ln_ were selected for the voxel-wise analysis of the [^18^F]HX4 uptake within the GTV, resulting in a Pearson’s correlation coefficient of 0.63 and 0.85, respectively. The HV during treatment largely overlapped within the HV at baseline (98 and 100 %, respectively; Fig. 3).

### Blood biomarkers

At baseline (*N* = 20) we measured an average concentration of 57 ± 26 ng/ml osteopontin, 190 ± 120 pg/ml CAIX, and 85 ± 67 pg/ml VEGF. There was no inter-correlation between the different plasma parameters. During (chemo)radiotherapy (*N* = 17), a non-significant decrease in CAIX (173 ± 97 pg/ml; *P* = 0.45) and VEGF (75 ± 67 pg/ml; *P* = 0.74) was observed, whereas the increase in osteopontin was significant (65 ± 31 pg/ml; *P* = 0.04; Supplementary Fig. 2).

### Relationship blood biomarkers and [^18^F]HX4 PET

At baseline none of the blood biomarkers (CAIX, VEGF, and osteopontin) showed a correlation with any of the [^18^F]HX4 PET image parameters. Also, no correlation between the blood biomarkers and the tumor volume was observed. During treatment, only the osteopontin concentration was weakly correlated with the SUV_mean_ on the [^18^F]HX4 PET (R_s_ = 0.52, *P* = 0.03; Supplementary Fig. 2).

## Discussion

In this study we evaluated tumour hypoxia with [^18^F]HX4 PET in patients with HNSCC before the start of (chemo)radiotherapy and during treatment, with the aim to monitor treatment response and evaluate the spatial variability of the [^18^F]HX4 uptake. In addition, the concentration of blood hypoxia markers (osteopontin, CAIX, and VEGF) was evaluated at baseline and during treatment. Last, the interdependence between hypoxia PET imaging and hypoxia-related blood biomarkers was investigated.

Before the start of (chemo)radiotherapy, we observed tumour hypoxia in the majority of primary HNSCC and metastatic lymph nodes. However, in most of these lesions, hypoxia disappeared during the course of treatment, regardless of the chosen treatment; radiotherapy alone or in combination with cisplatin or cetuximab. The decrease of hypoxia during treatment has been described in several studies [3, 19–21]. Lee et al. [19] showed with [^18^F]FMISO PET imaging a decrease in detectable tumour hypoxia from 90 % (18/20 patients) at baseline to 11 % (2/18) after 4 weeks of chemoradiotherapy. In addition, Servagi-Vernat et al. [20] observed a decrease of the [^18^F]FAZA PET uptake (SUV_max_ and HF) in all patients during chemoradiotherapy, which was recently confirmed by Bollineni et al. [21]. From our current results and the previous literature, we can conclude that in patients with HNSCC, hypoxia decreases during treatment, indicating tumour reoxygenation. Nevertheless, also a decrease in cell number, changes in vasculature, or perfusion could contribute to the altered hypoxia PET uptake.

Additionally, our results show that the patients with a high uptake at baseline have the highest chance of persistent hypoxia during treatment. These patients may benefit most from anti-hypoxia therapy during the entire course of treatment. For the other cases, the addition of anti-hypoxia therapy will probably have the largest therapeutic effect when given prior to, or during the first weeks of treatment. Since after this period, the amount of tumour hypoxia is low. [^18^F]HX4 PET imaging has already shown its potential to monitor the response to the anti-hypoxia treatment with TH-302 [22]. The ability to monitor the response to anti-hypoxia treatment with non-invasive imaging provides the potential to adapt the anti-hypoxia treatment based on the (changing) lesion characteristics.

Another frequently discussed method to target resistant tumour volumes is by giving a radiation boost. Previous studies have shown that it is technically feasible to provide a radiotherapy boost to hypoxic or metabolically active tumour subvolumes, defined on PET [4, 23–25]. For this purpose, information on the spatial repeatability of the hypoxia PET uptake and its stability during treatment is essential. In our current data-set only two lesions showed a significant amount of tumour hypoxia during treatment, with a spatially stable localization in comparison to the baseline scan. Where Bittner et al. [26] reported a geographically stable localization of the [^18^F]FMISO PET uptake during chemoradiotherapy, Nehmeh et al. [27], Lin et al. [27], and Servagi-Vernat et al. [20] reported changes in the distribution of hypoxia during treatment, and, therefore, adaptive radiotherapy based on serial hypoxia PET imaging was recommended.

Serial hypoxia PET imaging during treatment may provide additional information for response prediction. In a preclinical setting, micro-environmental parameters (hypoxia, perfusion) during treatment had a better potential to predict outcome after radiotherapy [28]. This finding was confirmed in a clinical study by Zips et al. [3] in 25 patients with HNSCC. The authors found a significant correlation between [^18^F]FMISO-PET imaging before and during chemoradiotherapy and local progression free survival, and tumour hypoxia during treatment was of higher prognostic relevance. In our study, accrual is ongoing and the assessment of hypoxia in relationship to outcome will be performed as data have matured.

We hypothesized that blood biomarkers could also be used to monitor treatment response, because previous studies have showed that blood biomarkers have the potential to predict response to treatment [29]. For example, a high concentration of plasma osteopontin was related to a higher amount of locoregional tumour failure in patients with HNSCC [14]. The blood biomarkers CAIX and VEGF, showed no significant changes during treatment and the osteopontin concentration was increased, while hypoxia PET imaging showed a clear reduction of the uptake in all patients. These results might be explained by the patient cohort. When comparing the plasma osteopontin levels of the patients in our current trial to the levels reported in the large randomized trial from Overgaard et al. [14], we observed that all our patients should be assigned to the low or intermediate concentrations of plasma osteopontin. Also, in comparison to a study in 295 patients with advanced rectal cancer, our observed levels of osteopontin and CAIX were lower than their reported average values [29]. In addition, plasma osteopontin is not only influenced by hypoxia, it is also known to play a role in the immune regulation and stress response [30]. An elevated level of osteopontin was for example observed in patients with systemic inflammatory response syndrome or sepsis [31]. The given anti-cancer treatment might have induced an immune/stress response causing an elevated level of osteopontin. The observed concentration of plasma CAIX (190 ± 120 pg/ml) was higher than documented for 209 patients with NSCLC (mean: 45 pg/ml) or 58 healthy individuals (mean: 2.5 pg/ml) [15]. The concentration of plasma VEGF (85 ± 67 pg/ml) was similar to that observed in patients with NSCLC [32] and higher than reported in 50 healthy women (median: 37 pg/mL [33]). Nevertheless, in the current population the blood biomarkers osteopontin, CAIX, and VEGF were not suitable to measure a hypoxia-related treatment response, which was measurable using [^18^F]HX4-PET.

The correlation between the blood hypoxia markers and the [^18^F]HX4 PET uptake was absent to weakly present. This was in agreement with the results previously published by several groups comparing hypoxia PET imaging to tissue or blood markers. Vercellino et al. [34] observed no correlation between the uptake of the hypoxia tracer [^18^F]FETNIM and the plasma osteopontin concentration in 16 patients with cervical carcinoma. In addition, Gronroos et al. [35] found no correlation between hypoxia PET imaging using FETNIM and several tissue biomarkers in 15 patients with HNSCC. While in patients with newly diagnosed glioma, the preoperative [^18^F]FMISO PET uptake was significantly, but weakly, correlated to the expression of VEGF in the tumour [36]. Although the current patient population is small, the results suggest that the tested blood biomarkers are not able to replace hypoxia PET imaging, or to pre-select patients for hypoxia PET imaging.

To conclude, hypoxia PET imaging with [^18^F]HX4 is able to detect tumour hypoxia in patients with HNSCC; in addition, it can monitor a decrease of tumour hypoxia during treatment. In patients with persistent tumour hypoxia, a stable localization of the hypoxic volume was observed. This provides potential for radiotherapy dose escalation to the hypoxic volumes. The blood parameters osteopontin, CAIX, and VEGF were not able to detect a decrease in hypoxia during treatment. In addition, there was no correlation between the blood plasma parameters CAIX and VEGF and hypoxia PET imaging, while only a weak correlation was observed between [^18^F]HX4 PET imaging and the osteopontin concentration during treatment. Based on the current data, we conclude that hypoxia PET imaging is the superior method to evaluate tumour hypoxia before and during treatment and cannot be replaced with the evaluated blood biomarkers.

## Electronic supplementary material

Below is the link to the electronic supplementary material.

Supplementary Table 1 [^18^F]HX4 PET/CT image derived parameters at baseline and during therapy; data was split based on the anti-cancer treatment. Shown are the mean, standard deviation, range, and the percentage difference of the baseline hypoxic lesions (GTV_prim_ and GTV_ln_), with an [^18^F]HX4 PET/CT scan at baseline and during treatment (total lesions: N=17). (DOC 37 kb)

Supplementary Fig. 1 Example of the regions of interest in the muscle tissue, which was used to calculate the tumor-background ratio. (GIF 592 kb)

High Resolution Image (TIF 4765 kb)

Supplementary Fig. 2 A: Observed increase in plasma osteopontin during treatment and B: significant correlation between the blood biomarker osteopontin, measured during treatment, and SUV_mean_ on the [^18^F]HX4 PET/CT. (GIF 27 kb)

High Resolution Image (TIF 31218 kb)
