# Supplementary material for: Evaluation of tumour hypoxia during radiotherapy using [18F]HX4 PET imaging and blood biomarkers in patients with head and neck cancer
Source: Eur J Nucl Med Mol Imaging. 2016 Jun 1;43(12):2139–46. doi: 10.1007/s00259-016-3429-y (PMC5047929; doi:10.1007/s00259-016-3429-y)
Supplement: Supplementary file 1 — [18F]HX4 PET/CT image derived parameters at baseline and during therapy; data was split based on the anti-cancer treatment. Shown are the mean, standard deviation, range, and the percentage difference of the baseline hypoxic lesions (GTVprim and GTVln), with an [18F]HX4 PET/CT scan at baseline and during treatment (total lesions: N=17). (DOC 37 kb) [file 259_2016_3429_MOESM1_ESM.doc]

Supplementary Table 1: [^18^F]HX4 PET/CT image derived parameters at baseline and during therapy; data was split based on the anti-cancer treatment. Shown are the mean, standard deviation, range and the percentage difference of the baseline hypoxic lesions (GTV_prim_ and GTV_ln_), with an [^18^F]HX4 PET/CT scan at baseline and during treatment (total lesions: N=17).

|  | **Baseline** | **During treatment** | **Difference [%]** |
| --- | --- | --- | --- |
| **Cisplatin chemo-radiotherapy (N=10)** |  |  |  |
| TMR_max_ | 2.0±0.5  (1.4-2.8) | 1.4±0.3  (1.0-2.1) | -29±12 |
| Hypoxic fraction [%] | 28±24  (3-71) | 6±13  (0-40) | -91±19 |
| Hypoxic volume [cm^3^] | 27.8±23.6  (2.9-70.5) | 1.4±3.2  (0.0-10.1) | -91±19 |
| **Radiotherapy (N=4)** |  |  |  |
| TMR_max_ | 1.9±0.3  (1.7-2.3) | 1.4±0.1  (1.3-1.5) | -23±13 |
| Hypoxic fraction [%] | 14±5  (7-18) | 1±1  (0-2) | -94±6 |
| Hypoxic volume [cm^3^] | 3.5±1.9  (0.7 -5.1) | 0.2±0.3  (0.0-0.6) | -94±6 |
| **Cetuximab-radiotherapy (N=2)** |  |  |  |
| TMR_max_ | 1.7±0.4  (1.5-2.1) | 1.3±0.2  (1.2-1.5) | -25±5 |
| Hypoxic fraction [%] | 12±12  (3-25) | 0.2±0.3  (0.0-0.5) | -99±1 |
| Hypoxic volume [cm^3^] | 1.1±1.1  (0.4-2.4) | 0.0±0.0  (0.0-0.1) | -99±1 |
