# Supplementary figures and images for: Evaluation of tumour hypoxia during radiotherapy using [18F]HX4 PET imaging and blood biomarkers in patients with head and neck cancer
Source: Eur J Nucl Med Mol Imaging. 2016 Jun 1;43(12):2139–46. doi: 10.1007/s00259-016-3429-y (PMC5047929; doi:10.1007/s00259-016-3429-y)

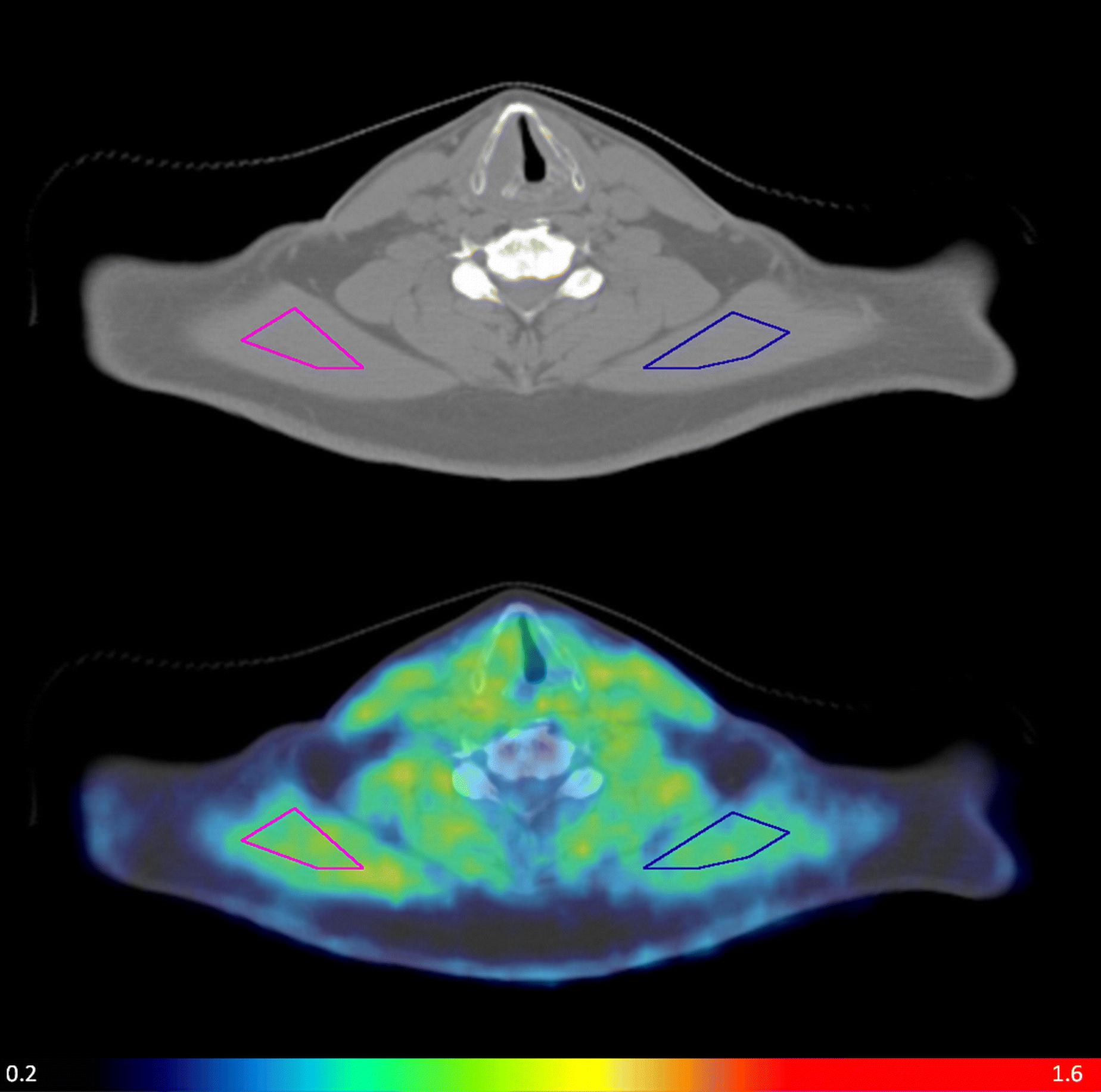

Supplement: Supplementary file 2 — Example of the regions of interest in the muscle tissue, which was used to calculate the tumor-background ratio. (GIF 592 kb) [file 259_2016_3429_Fig4_ESM.gif]

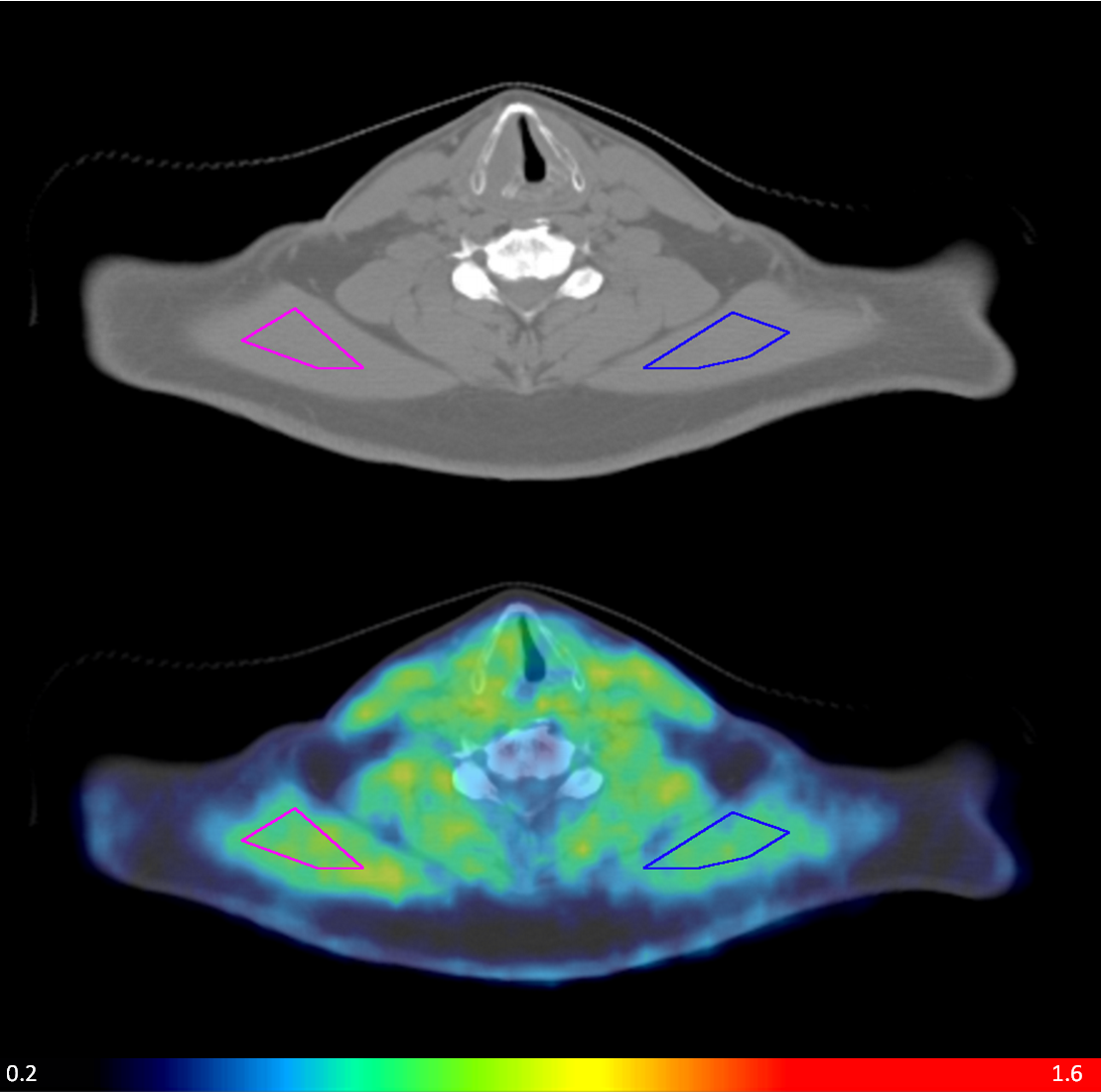

Supplement: Supplementary file 3 — High Resolution Image (TIF 4765 kb) [file 259_2016_3429_MOESM2_ESM.tif]

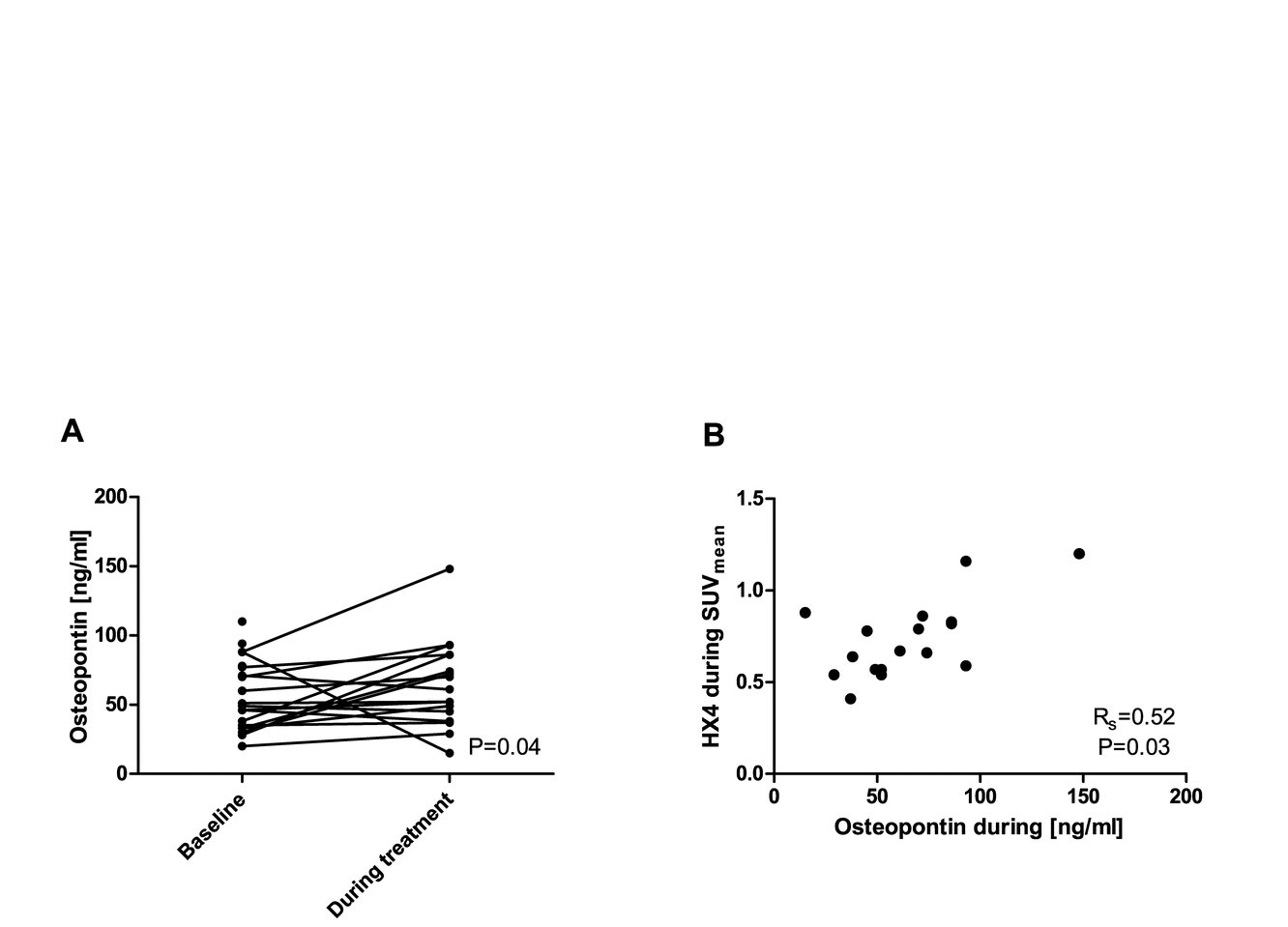

Supplement: Supplementary file 4 — A: Observed increase in plasma osteopontin during treatment and B: significant correlation between the blood biomarker osteopontin, measured during treatment, and SUVmean on the [18F]HX4 PET/CT. (GIF 27 kb) [file 259_2016_3429_Fig5_ESM.gif]

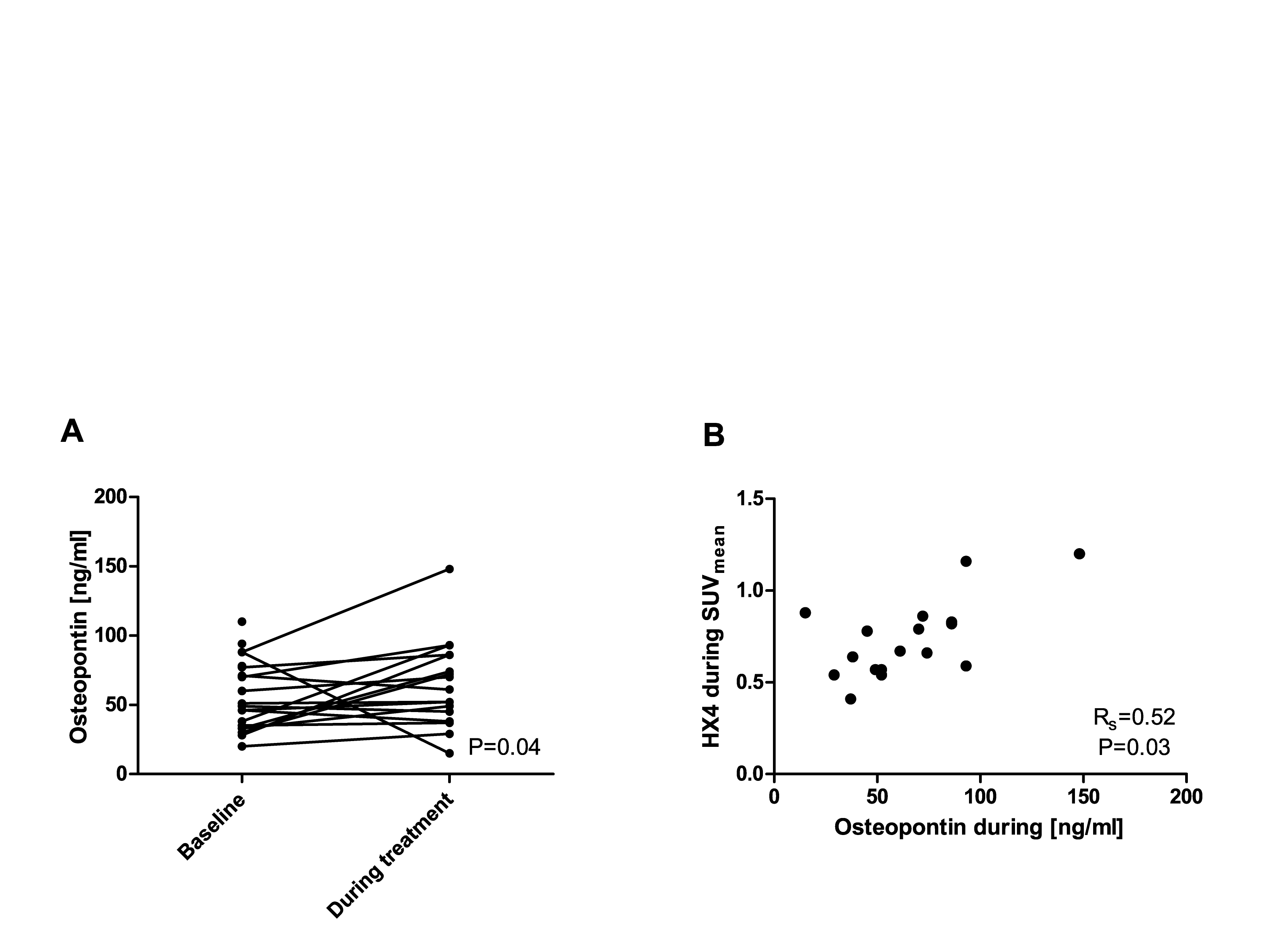

Supplement: Supplementary file 5 — High Resolution Image (TIF 31218 kb) [file 259_2016_3429_MOESM3_ESM.tif]
